# Long-Term and Meditation-Specific Modulations of Brain Connectivity Revealed Through Multivariate Pattern Analysis

**DOI:** 10.1007/s10548-023-00950-3

**Published:** 2023-03-28

**Authors:** Roberto Guidotti, Antea D’Andrea, Alessio Basti, Antonino Raffone, Vittorio Pizzella, Laura Marzetti

**Affiliations:** 1 Department of Neuroscience, Imaging and Clinical Sciences, “Gabriele d’Annunzio” University Chieti- Pescara, Via dei Vestini 33, 66013 Chieti, Italy; 2 Institute for Advanced Biomedical Technologies, “Gabriele d’Annunzio” University Chieti-Pescara, 66013 Chieti, Italy; 3 Department of Psychology, “La Sapienza” University Rome, 00185 Rome, Italy

**Keywords:** FMRI, Functional connectivity, Machine learning, Focused attention mediation, Open monitoring meditation, Mindfulness

## Abstract

Neuroimaging studies have provided evidence that extensive meditation practice modifies the functional and structural properties of the human brain, such as large-scale brain region interplay. However, it remains unclear how different meditation styles are involved in the modulation of these large-scale brain networks. Here, using machine learning and fMRI functional connectivity, we investigated how focused attention and open monitoring meditation styles impact large-scale brain networks. Specifically, we trained a classifier to predict the meditation style in two groups of subjects: expert Theravada Buddhist monks and novice meditators. We showed that the classifier was able to discriminate the meditation style only in the expert group. Additionally, by inspecting the trained classifier, we observed that the Anterior Salience and the Default Mode networks were relevant for the classification, in line with their theorized involvement in emotion and self-related regulation in meditation. Interestingly, results also highlighted the role of specific couplings between areas crucial for regulating attention and self-awareness as well as areas related to processing and integrating somatosensory information. Finally, we observed a larger involvement of left inter-hemispheric connections in the classification. In conclusion, our work supports the evidence that extensive meditation practice modulates large-scale brain networks, and that the different meditation styles differentially affect connections that subserve style-specific functions.

**Supplementary Information:**

The online version contains supplementary material available at 10.1007/s10548-023-00950-3.

## Introduction

The growing interest in exploring the fundamental facets of meditation is driven by its beneficial effects on several cognitive and psychological processes, in the framework of mental and physical health promotion (Lutz et al. 2008; Tang et al. 2015).

Meditation practices have been divided into three macro-families, i.e. attentional, deconstructive and constructive (Dahl et al. 2015). Among these families, attentional meditation is known to strengthen the self-regulation of different attentional processes, as well as the ability to initiate and sustain meta-awareness. The neural underpinnings of these cognitive processes have been investigated by further disentangling two main styles of attentional meditation: the focused attention (FA), in which the core is to narrow the attention on a specific endogenous object (e.g. breath sensations), and the open monitoring (OM), where the attentional scope is expanded to detect emotional feelings, thoughts and perceptions as they occur (Lutz et al. 2008; Vago and Zeidan 2016).

Recent studies demonstrated that both FA and OM meditation styles modulate the activity and connectivity of different brain regions and networks devoted to the regulation of attention, self-awareness and self-monitoring, inhibitory control, shaping the communication between brain areas using EEG (Cahn and Polich 2006; Yordanova et al. 2020), fMRI (De Filippi et al. 2022; Manna et al. 2010) and MEG (Calvetti et al. 2021; Marzetti et al. 2014).

Although several studies have focused on how specific meditation styles modulate the structural and functional aspects of the human brain (Ganesan et al. 2022; Pernet et al. 2021; Tang et al. 2015), the gap between the phenomenology of meditation and the underpinning neural mechanisms still needs to be filled. Indeed, it is still unclear how different meditation styles and extensive practice impact large scale brain networks, and their intra- and inter-hemispheric connections.

In this scenario, the ability of decoding approaches, also referred as multivariate pattern analysis (MVPA) to detect fine-grained differences in neurophysiological signals (O’Toole et al. 2007) is suited for addressing this issue (Haxby 2012; Haynes 2015). Indeed, it has been demonstrated that MVPA can be successfully used to discriminate patterns of brain activation in perceptual and cognitive tasks (Cichy et al. 2014; Guidotti et al. 2020; Kragel and LaBar 2016; Tosoni et al. 2016). The same approach has been used by exploiting functional connectivity patterns to decode discrete functions such as working memory (Syrjälä et al. 2021) or continuous variables such as brain maturity or age (Dosenbach et al. 2010; Liem et al. 2017). Moreover, MVPA has been applied in mindfulness studies to predict age using voxel-based morphometry (Luders et al. 2016) and to classify patterns of functional connectivity before and after a body-mind training course (Tang et al. 2017). Recently, different signatures of age and expertise on patterns of functional connectivity in expert meditators have been shown (Guidotti et al. 2021).

In this study, we analyze fMRI data recorded from two groups of subjects, with different levels of meditation expertise, while performing both FA and OM meditation styles. Relying on a connectivity based MVPA approach, we investigate whether functional connectomes are predictive of style-specific meditation state, and if the meditation expertise can impact the decoding accuracy. Finally, the intra- and inter-hemispheric large-scale connectivity patterns relevant for the prediction are explored. Based on proposed theories and earlier neuroimaging findings (Lutz et al. 2015; Manna et al. 2010; Marzetti et al. 2014; Raffone et al. 2019), we hypothesize a particular involvement of connectivity within and between default mode, salience and central executive networks, as well as a prominent involvement of connectivity within the left hemisphere.

## Materials and Methods

### Participants

Our study involved twelve Theravada Buddhist monks (males, mean age 37.9 yrs, SD 9.4 yrs, range 25–53 years) from the Santacittarama Buddhist Monastery. The participants followed a Thai Forest Tradition and practiced FA (Samatha) and OM (Vipassana) meditation forms in a balanced way, including silent meditation retreats (3 or 4 months per year). Meditation expertise is measured in years since the beginning of meditation practice in the monastic context (mean 16.4 yrs, SD 7.7 yrs). A group of ten novice meditators (males, mean age 33.0 yrs, SD 4.0 yrs, range 26–36 years) were also recruited from the local community. The subjects included in the novice group practiced both styles, 30 minutes each day, for 10 days supervised by an expert meditator, before the fMRI acquisition. The participants gave their written informed consent according to the Declaration of Helsinki and the study was approved by the Ethical Committee of the University of Chieti-Pescara.

### Experimental Procedure

**Fig. 1:**
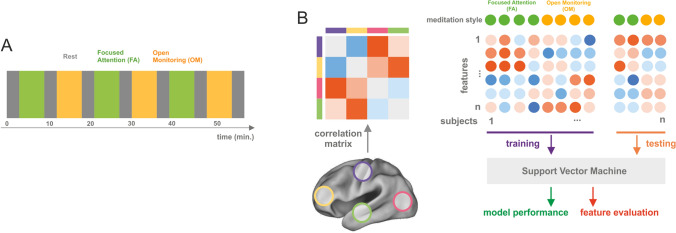
Experimental paradigm and analysis schema. Panel A the experimental procedure consisted of a block paradigm in which 6 min. FA meditation, 6 min. OM meditation intermixed with a 3 min. non-meditative resting state were repeated three times. Panel B the analysis pipeline consists in extracting the average time course of the preprocessed BOLD signal (see "Materials and Methods" section) from 90 ROIs, and in computing the pairwise Pearson correlation matrix between the extracted time courses; then, the correlation matrix is used for training a Support Vector Machine (SVM) and predicting the meditation style. Before training, an ANOVA-based feature selection is performed. The dataset is randomly split 200 times into two parts: 75% of the subjects are used for training and 25% for testing. Finally, accuracy is calculated to assess the model performance and the importance of features is evaluated by extracting the frequency of selection of a feature and by inspecting the weights of the model

The experiment alternated a block of 6 minutes of FA meditation and a block of 6 minutes of OM meditation. Each meditation block was preceded by 3 minutes of non-meditative passive block (Fig. 1A). The subjects performed three blocks of FA meditation and three blocks of OM meditation for a total duration of 57 minutes.

Each block started with verbal instructions on the mediation style to be performed or on resting. For FA meditation, the following instruction were given: “gently engage in sustaining the focus of your attention on breath sensations, such as at the nostrils, noticing with acceptance and tolerance any arising distraction, as toward stimuli or thoughts, and return gently to focus attention on the breath sensations after having noticed the distraction source”. In OM meditation, participants were given the following instruction: “observe and recognize any experiential or mental content as it arises from moment to moment, without restrictions and judgment, including breath and body sensations, percepts of external stimuli, arising thoughts and feelings”. The instruction for Rest was the following: “rest in a relaxed awake state”.

### Data Acquisition

Data were acquired by a 1.5 T Siemens Magnetom Vision Scanner, BOLD signal images were obtained using a T2*-weighted echo planar (EPI) sequence with: TR = 4.087 s, 28 slices and voxel size 4 mm × 4 mm × 4 mm for a total of 860 functional volumes. A high-resolution T1-weighted whole-brain image was also acquired at the end of each session via a 3D-MPRAGE sequence (sagittal matrix = 256 × 256, FOV = 256 mm, slice thickness = 1 mm, no gap, in-plane voxel size = 1 mm × 1 mm, flip angle = 12°, TR/TE = 9.7/4.0 ms).

### Preprocessing

Preprocessing of fMRI was carried out using the same approach used in our previous papers (Guidotti et al. 2021; Manna et al. 2010): the first five volumes were dropped, then motion and temporal correction was applied. Data were then filtered using a high-pass filter of two cycles per time course and then a linear detrend was applied. We did not include spatial and temporal smoothing since it affects connectivity patterns (Alakörkkö et al. 2017). The standard preprocessing was performed using Brain Voyager QX 1.7 software (Brain Innovation, Netherlands).

Then, we segmented anatomical images into grey matter, white matter, and cerebrospinal fluid (CS) using the FSL FAST algorithm (Zhang et al. 2001). White-matter and CSF average timecourses were regressed out from the fMRI signal, together with movement parameters. The residual was filtered (0.009–0.08 Hz) and finally scrubbed by removing the volumes with a Framewise Displacement (FD) index greater than 0.5 (Power et al. 2012).

### Regions of Interest (ROI)

To investigate the role of brain networks in distinguishing between FA and OM meditation styles, we adopted the functional ROIs parcellation introduced in (Shirer et al. 2012) consisting in a set of ROIs that broadly covers a large part of the cerebral cortex using a functional subdivision. The authors extracted 90 ROIs from 14 networks obtained with ICA from resting state fMRI data: Visuospatial, High, and Primary Visual, left, and right Executive Control, posterior, and anterior Salience, dorsal, and ventral Default Mode, Sensorimotor, Basal Ganglia, Language, Auditory, and Precuneus networks. The selected ROI list is presented in Table S1.

### Functional Connectivity Analysis

The average ROI time course was extracted and Pearson's correlation coefficient between time courses was computed from the ROIs obtained for each subject, independently for each meditation modality, and for each experimental block. Then, Fisher's z-transformation was applied and a square correlation matrix for each block was obtained.

### Decoding Analysis

Values in the upper triangle of the correlation matrix were used as features to feed the classifier (Fig. 1B). Specifically, we extracted the feature set for each participant, block, and meditation style. Therefore, our dataset for classification was composed of six correlation matrices per participant divided in two classes, for a total of 36 samples for experts (3 matrices by 12 participants) and 30 samples (3 matrices by 10 participants) for novices.

An ANOVA-based feature selection (Pereira et al. 2009) was applied to reduce the dataset dimensionality, since the ratio between the number of samples and features was very large. We selected the 200 highest F-score ranked features.

We performed model selection by means of cross-validation. The dataset was then split into two parts: 75% of samples were used as a training set and the remaining 25% as testing set. This procedure was repeated 200 times, by shuffling samples in the training set to provide a good estimator of the prediction error. Feature selection was only applied to the training set for each repetition, in order to avoid biasing of the prediction error (Pereira et al. 2009).

We then analyzed the dataset using a linear SVM (with penalty coefficient C = 1) and we computed the classification accuracy to evaluate model performance. All these analyses were carried out with the *scikit-learn* (Pedregosa et al. 2011), *nilearn* (Abraham et al. 2014) and *scipy/numpy* packages (Virtanen et al. 2020).

### Relevant Feature Analysis

The ANOVA-based feature selection procedure extracts the connections that will be used to train the classifier; this procedure is repeated for each cross-validation fold.

First, we computed the feature selection probability by dividing the selection frequency by the number of cross-validation folds (n = 200), then we averaged the selection probability for within- and between-network connections, in order to understand the contribution of each single network to the classification.

The contribution of each connection was then obtained by thresholding the feature selection probability; we selected only the connections that were selected in all of the cross-validation folds, thus those having probability equal to 1 of being selected.

Finally, we extracted the weights of the classifier only for the most selected connections to understand the specific contribution of the connection to each meditation style.

### Statistical Analysis

Permutation test was used to assess the statistical significance of the decoding accuracy (Nichols and Holmes 2003): condition labels were shuffled two hundred times (n = 200) and the classification algorithm was repeated, including feature selection and cross-validation, to obtain the null distribution for the classification accuracy. The reported p-value is the ratio between the number of permutations that outperform the case with no permuted labels and the number of permutations. The p-value is the probability of observing the reported error by chance using the null distribution obtained after permutations. Multiple comparisons were assessed using Bonferroni correction.

We used a Mann-Whitney *U* test to compare the distribution of accuracies in the two groups. In addition, we carried out a Bayesian hypothesis testing approach to compare the significance or the two classifiers with a null (or dummy) classifier (Benavoli et al. 2017).

### Control Analyses

We performed two control analyses to check whether the sample size and the age affected the decoding accuracy in the two groups.

In order to check the impact of the limited sample size in the decoding accuracy (Cui 2018, Varoquaux 2018), we performed a bootstrap analysis on the expert group. In particular, we resampled (n = 100), without replacement, the expert dataset (n. subjects = 12) to the size of the novice (n. subjects = 10) and tested how the accuracies are distributed.

The different age of the two groups may introduce a bias in the classification accuracy. A possible solution could be to regress the age out from the datasets and use the residuals for the classification (Bron et al. 2015; Snoek et al. 2019), although this procedure may remove some variance related to the classification task. We followed this approach and performed, in both groups, the same classification analysis with a dataset cleaned by the age variable.

## Results

### Connectivity Patterns Predict Meditation Style in Experts Meditators

The classification analysis aimed at decoding the meditation style from functional connectomes in the two groups of subjects, novice and expert meditators, separately.

Our results show that the prediction accuracy of the meditation style was equal to 65.6% for the expert group (p = 0.01; permutation-test, n = 200, avg. accuracy = 41.4%; Bonferroni-corrected), and to 54.4% (p = 0.14; permutation-test, n = 200, average accuracy = 41.03%; Bonferroni-corrected) for the novice group. We used a Mann-Whitney *U* test to check whether the distribution of cross-validation accuracies in the expert group was higher than in the novice group. The test showed that the accuracies in the expert group were significantly higher than those in the novice group (U = 25.263e + 3, n_1_ = n_2_ = 200; p < 0.001 two-sided).

The Bayesian hypothesis test showed that the accuracy distribution in the expert dataset was significantly different from the null-model (p < 0.001), while in the novice group the difference is not significant (p = 0.33).

These findings thus reveal that the large-scale functional interactions can be more accurately used to predict the meditation style in expert monks than in the novice group.

We tested whether the age and the sample size seems to affect the decoding accuracy.

The bootstrapping analysis, used to check the effect of the sample size, showed that the average accuracy of the resampled expert dataset was equal to 63.1% (p = 0.02 Bonferroni-corrected). By comparing the bootstrapped accuracy distribution with the original values, we found that novice accuracy was at the 4-th percentile, while it was at 58-th percentile for the experts. These results suggested that the difference in the sample size has no effect on the accuracies.

Control analysis for the age confirmed previous findings. Specifically, we significantly decoded the meditation form in experts (avg. accuracy = 62.9%, p = 0.02 Bonferroni-corrected) while the accuracy was not significant in the novice group (accuracy = 53.7%, p = 0.1 Bonferroni-corrected). Although the decoding accuracy slightly decreased for both groups, the age does not seem to play a role in the accuracy difference.

### Salience and Default Mode Networks are the Most Relevant for Meditation Style Decoding

In order to understand which brain networks, as described in (Shirer et al. 2012), play a crucial role in the decoding of meditation styles, we inspected the feature set used by the expert meditators classification model.

We calculated the frequency of a selected feature by evaluating the number of times that a single feature was used for classification, and we obtained a selection probability matrix by dividing the frequency of each feature by the number of cross-validation folds (n = 200). We then extracted the network selection probability by averaging the probabilities of the between- and within-network connectivities, thus it indicates the probability that a node belonging to that particular network is selected. We checked the F-statistics of the relevant features and we found that all the reported variables had a statistically significant difference between the two conditions (min: F(1, 52) = 4.493; p = 0.038). Moreover, we compared the distribution of the weights in the cross-validation fold by testing whether the negative and positive distributions were statistically different from zero, using a 1-sample *t*-test. We found that all the relevant features were significantly different from 0 (dof = 199, p < 0.001; Bonferroni-corrected)

**Fig. 2:**
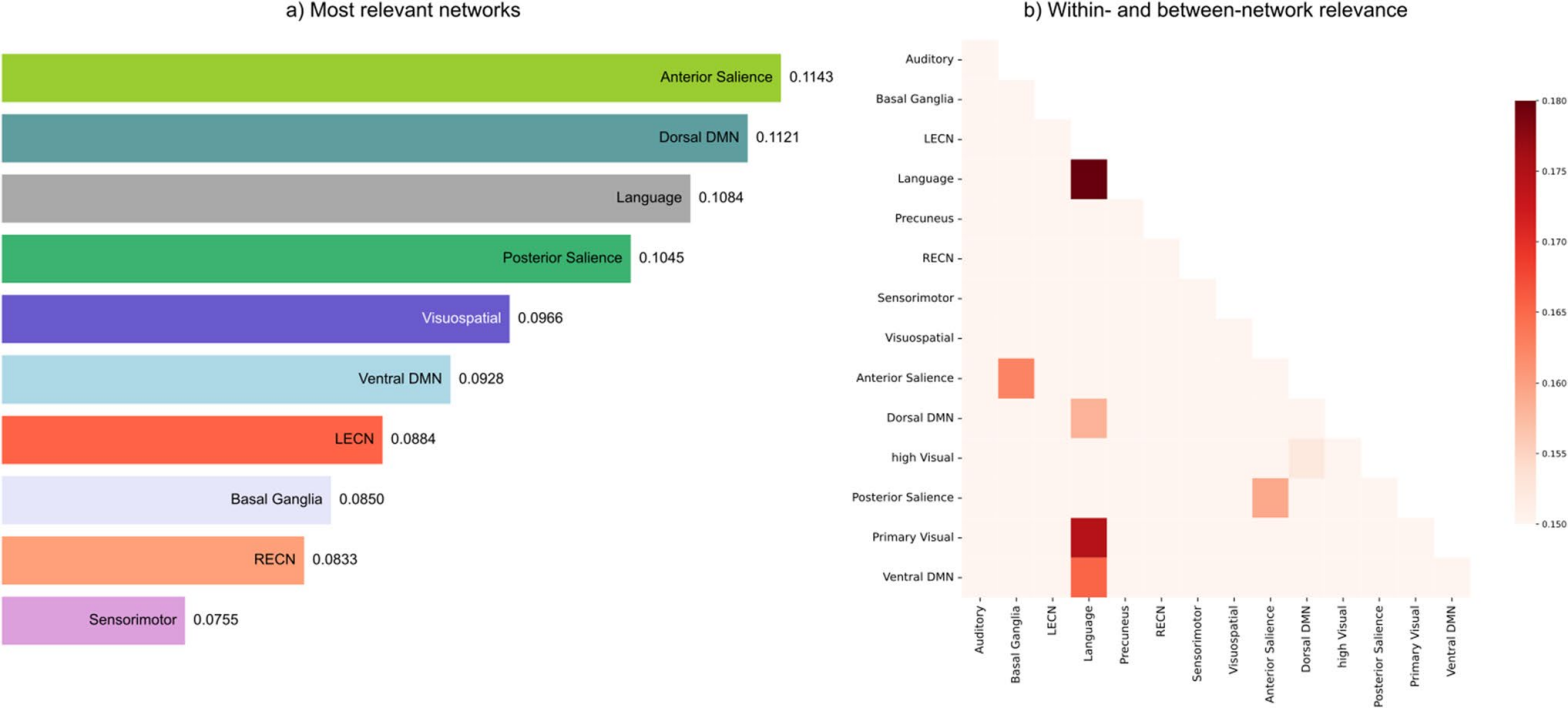
Relevant networks for meditation style decoding. The contribution of a network is measured as the average selection probability of both between- and within- network connections. Panel A) The bar plot shows the relevance of the ten most important networks. Panel B) The plot illustrates the between- and within- network relevance. Each square represents the relevance of the between- and within-network connections calculated as the average selection probability. List of abbreviations: DMN - Default Mode Network; LECN - left Executive Central Network; RECN - right Executive Central Network

In Fig. 2A, the barplot shows the ten networks with the highest probability of being selected for the classification. The results highlight that both the Anterior Salience network and the dorsal Default Mode network (DMN) are the most relevant networks involved in the discrimination between meditation styles in the expert group (Fig. 2A), while the right Executive Central (RECN) and sensorimotor networks are less prone to predict the meditation style. Moreover, by disentangling the within- and between-network contributions, we observed that the Language network plays an important role as shown in Fig. 2B. This network, indeed, shows within-network interactions as well as interactions with the ventral DMN and the primary visual network, crucial for the meditation style decoding. Our findings also show a relevant coupling between the anterior and the posterior Salience network and Basal Ganglia and the anterior Salience network.

### Specific Connections Subserve Different Meditation Styles

To investigate the contribution of each meditation-specific inter-areal connection, we thresholded the selection probability matrix, and then we extracted the weights of the most probable features averaged across cross-validation folds.

**Fig. 3:**
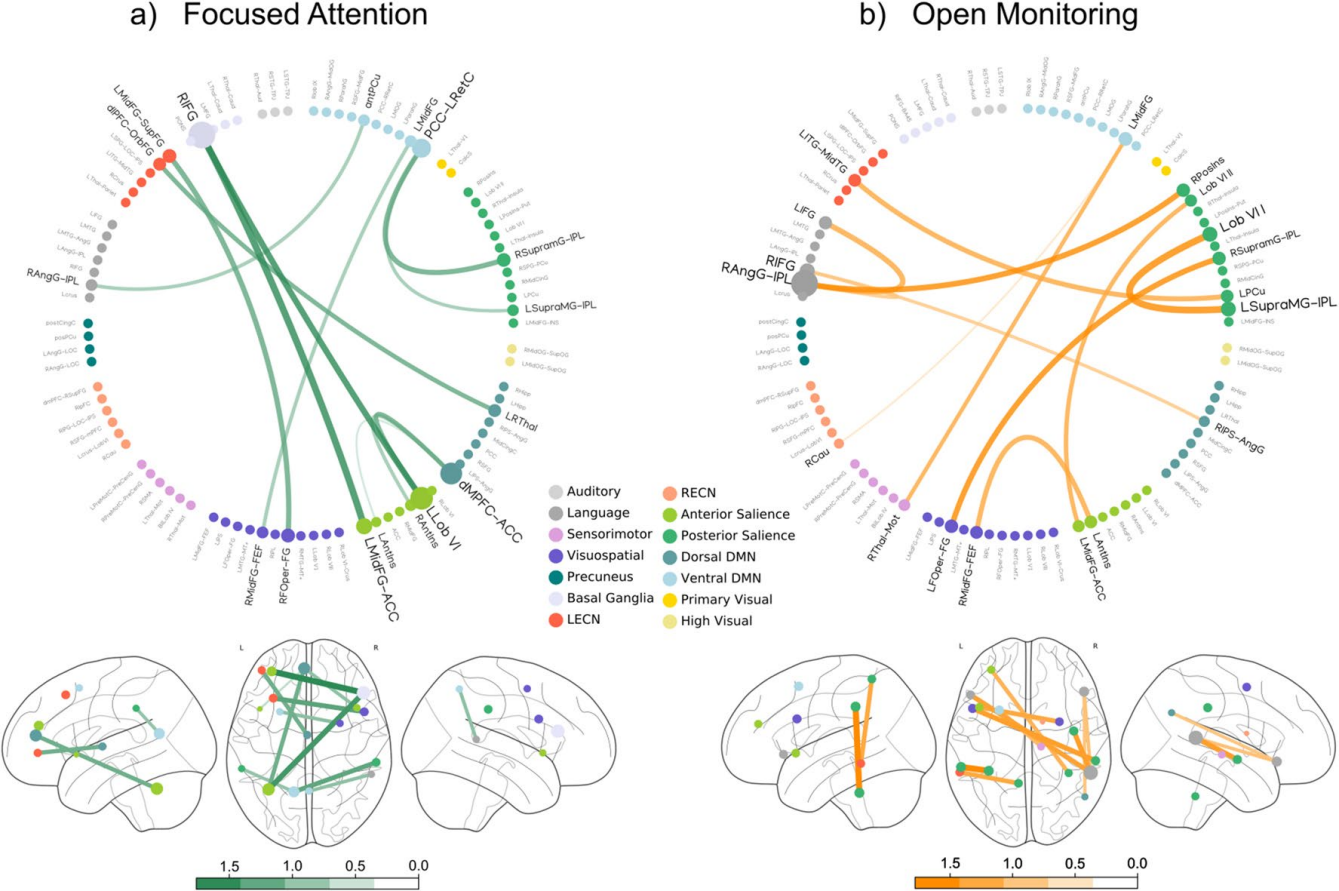
Meditation-specific weights of the style decoding model

We separated the connections with positive weights, specific to FA meditation (Fig. 3A), from those with negative weights specific to OM meditation (Fig. 3B). Line thickness and color hue indicate the weight magnitude.

Both graphs highlight that the contribution of cerebellar connections, including those of the left declive areas (Left Lobule VI), results to be significant for both FA and OM meditation styles (Fig. 3A–B). More in detail, Fig. 3A shows the interactions specific for the FA meditation style: the connections between the right and the left supramarginal gyri seem to play a relevant role. Importantly, these areas are parts of a high-level system used to process somatosensory, auditory and visual stimuli (Tomasino et al. 2012), comprising the angular gyrus (AngG), and the inferior parietal lobule (IPL). Moreover, the right AngG-IPL is coupled with the right posterior insula and the left inferior frontal gyrus, suggesting the presence of a circuit sharing information among these regions. Additionally, our results shows that Focused Attention (FA) specific connections include the anterior cingulate cortex (ACC), the medial prefrontal cortex (mPFC) and the posterior cingulate cortex (Fig. 3A), which are involved in attentional control and self-awareness.

The figure shows the normalized feature weights associated with the meditation style prediction model averaged across cross-validation folds. These weights are specific for FA (Panel A) and OM (Panel B) meditation styles. Each ROI is represented by a circle, whose size is proportional to the average of the weights.

### Large-Scale Core Networks Orchestrate Within- and Between-Hemispheric Communication in Meditation

In order to understand the contribution of the left and right hemispheres, we analyzed the distribution of the most relevant intra- and inter-hemispheric connections. In particular, we tested whether the left-hemispheric weights were higher than the right-hemispheric. Using a Mann-Whitney *U* test, we found that the magnitude of left-hemispheric weights was higher than those in the right hemisphere (U = 510.e + 3; n_1_ = 946, n_2_ = 1035, p < 0.05 one-sided).

**Fig. 4:**
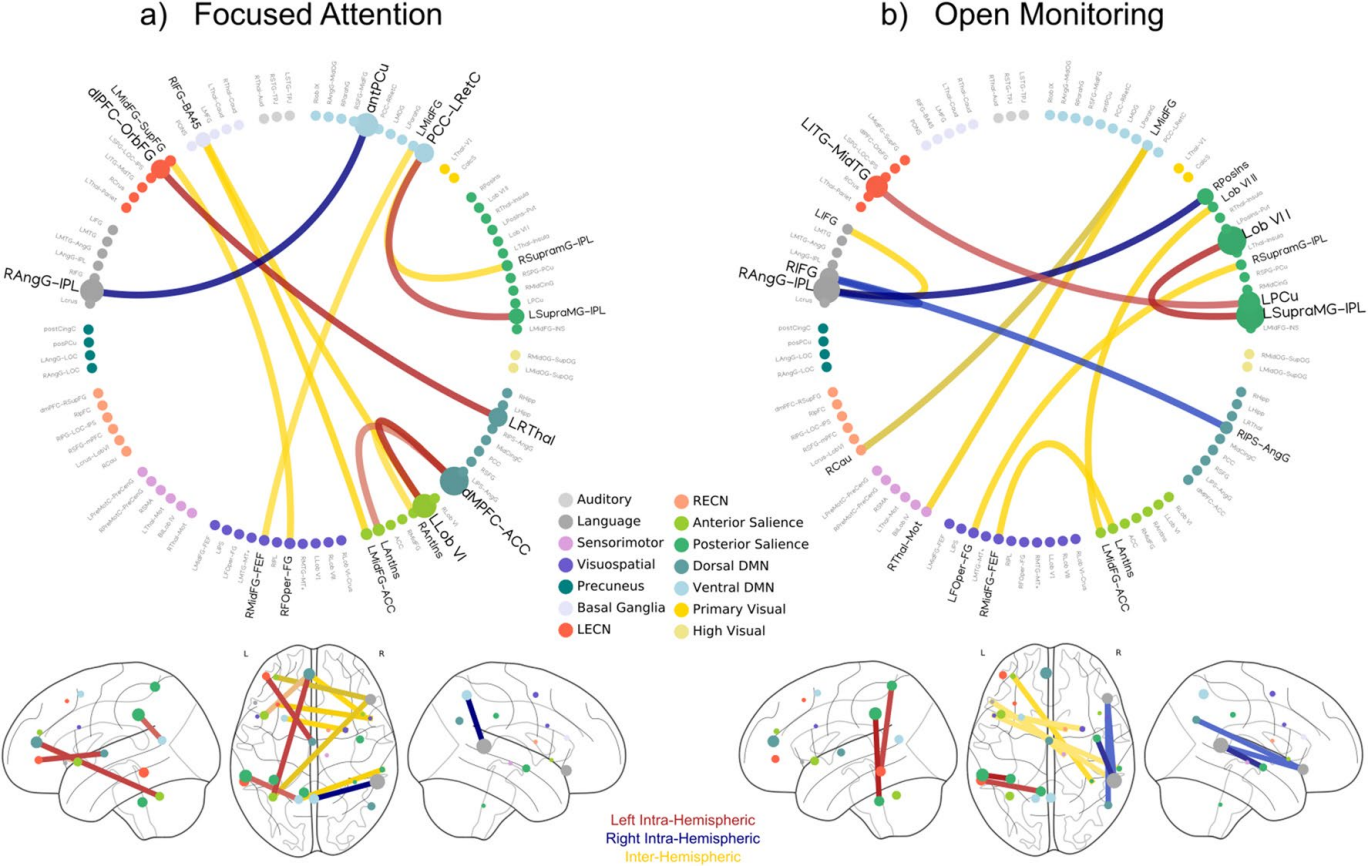
Intra-and inter- hemispheric relevant connections. Red lines represent the left hemisphere connections, while the right hemisphere connections are denoted by blue lines. Finally, the inter-hemispheric connections are depicted by yellow lines

Figure 4 shows a higher number of relevant intra-hemispheric connections in the left hemisphere with respect to the right., although the number of connections in the leftward part of the brain was not significant (n = 6, p = 0.055; permutation test), as well as in the rightward part (n = 4; p = 0.28; permutation test). Qualitatively, in the left hemisphere, the results show an interplay between the Executive, the Salience and the Default Mode networks, while in the right hemisphere key nodes of the Auditory, dorsal Default Mode and posterior Salience networks are involved. Furthermore, Fig. 4 also shows the contribution of nodes included in the frontal lobe in the inter-hemispheric integration of information.

Taken together, these findings support the foundational theories of the left-dominance of intra-hemispheric connectivities and the involvement of frontal intra-hemispheric regions for the integration and processing of information.

## Discussion

It is well established that meditation practice effectively shapes the communication between brain areas and networks thus regulating different cognitive and behavioral processes, such as attention, self-monitoring and inhibitory control (Cahn and Polich 2006; Lutz et al. 2015; Manna et al. 2010; Marzetti et al. 2014).

In this scenario, a reliable tool to decode fine-grained connectivity patterns modulated by meditative practice is represented by multivariate pattern analysis (MVPA). In the present study, by using a machine learning approach, we demonstrated how different large-scale functional connectivity patterns underpin different meditation styles. In particular, we showed that the prediction accuracy is significantly higher in the expert group than in the novice group, whose connectivity profiles are not informative about the meditative specialization. These findings suggest that the expertise in meditation practice shapes and consolidates specific neural patterns subserving different meditation styles. By predicting meditation-style across-subjects, our analysis demonstrated the existence of a shared trace of long-term meditation practice in brain functional connectivity. Specifically, this common trace is represented by patterns of coupled nodes belonging to different networks relevant for the decoding.

The results highlighted that a crucial role for meditative style classification is played by two networks largely described as strongly involved in interoceptive and self-related processes: the Salience and Default Mode networks (Melis et al. 2022; Raffone et al. 2019). Conversely, the Executive network, which is classically defined as a core system for coordinating attentional processes in the meditating brain (Bauer et al. 2019; Doll et al. 2015), seems to be less prone to predict the meditation style. Interestingly, also the Language network and the Visual network revealed to be important to decode OM and FA meditation styles. Conceivably, the role of the former may be related to simple verbal labeling of the internal and external perceived stimuli naturally occurring during OM meditation (Lutz et al. 2008; Raffone et al. 2019; Vago and Zeidan 2016), while the role of the latter may be related to its importance in regulating the attentional resources during meditative visual mental imagery (Raffone and Srinivasan 2009).

By inspecting connections relevant for the prediction, we found the contribution of the left declive areas (Left Lobule VI) of the cerebellum that are common for both FA and OM. Indeed, an increase of cerebellum gray matter density and volume has been found in long-term meditators (Vestergaard-Poulsen et al. 2009), and in novice practitioners after a 8-week mindfulness-based training (Hölzel et al. 2011), possibly related to the different roles of this area in the regulation of the posture and of the emotional level (Levisohn et al., 2000; Park et al. 2018). In addition, the coupling of left declive with the right supramarginal gyrus, revealed by our analysis, might be related to the meditators ability of being empathetic and compassionate (Lippelt et al. 2014; Lutz et al. 2008), given the role of the right supramarginal areas in empathy regulation (Silani et al. 2013).

Our results showed that Open Monitoring (OM) specific connections involve the angular gyrus (AngG), and the inferior parietal lobule (IPL), two regions that are supposed to process somatosensory, auditory and visual stimuli (Numssen et al. 2021; Seghier 2013). In addition, the right AngG-IPL is coupled with the right posterior insula and the left inferior frontal gyrus, suggesting the presence of a circuitry used to process and label the current emotional status (Seghier 2013).

Focused Attention (FA) specific connections, instead, include nodes that are involved in attentional control and self-awareness, as the frontal eye field (FEF), the anterior cingulate cortex (ACC), the medial prefrontal cortex (mPFC) and the posterior cingulate cortex (PCC). In particular, the ACC is known to have a strong relevance in the self-regulation of attention and conflict monitoring (Posner and Rothbart 2007; Van Veen et al. 2009) and has been demonstrated to increase its activity during FA meditation in experienced meditators rather than in novices (Zhang et al. 2021).

In addition, the modulation of PCC connections, due to extensive meditation, is well established (Brewer and Garrison 2014; Marzetti et al. 2014; Zhang et al. 2021), due to its role in potentially switching between mind-wandering and focused attention.

In line with previous findings (Manna et al. 2010; Marzetti et al. 2014), our results also showed a left hemispheric dominance (Fig. 4); indeed the interplay between the left Salience, Default Mode and Executive networks supports the notion that meditation-related mechanisms are used to improve a rapid regulation and integration of information from different systems (Raffone et al. 2019). Instead, right connections, which involve the Auditory, the dorsal Default Mode and the posterior Salience networks, may be relevant for accumulating or integrating interoceptive and emotional information, subsequently labeled by the Language network during OM style ((Bud) Craig 2009).

In addition, we also showed that relevant inter-hemispheric connections include those with nodes in the frontal lobe, a region that showed a structural change in expert mediators as reported in previous studies (Luders et al. 2011; Tang et al. 2012).

Overall, our results suggest that style-specific connectivity fingerprints emerge from extensive meditative practice. This shared trace is characterized by patterns of both within- and between- left dominant connectivity networks which are modulated by meditation-specific cognitive and behavioral processes.

## Supplementary Information

Below is the link to the electronic supplementary material.

Supplementary material 1 (DOCX 11.3 kb)

## Data Availability

The datasets generated during and/or analysed during the current study are available from the corresponding author on request.
