# Supplementary material for: Long-Term and Meditation-Specific Modulations of Brain Connectivity Revealed Through Multivariate Pattern Analysis
Source: Brain Topogr. 2023 Mar 28;36(3):409–18. doi: 10.1007/s10548-023-00950-3 (PMC10164028; doi:10.1007/s10548-023-00950-3)
Supplement: Supplementary file 1 — Supplementary material 1 (DOCX 11.3 kb) [file 10548_2023_950_MOESM1_ESM.docx]

******************************* Table S1 *****************************************

| **Coordinates** | | | **Regions** | **Labels** | **Network** | **Voxels** |
| --- | --- | --- | --- | --- | --- | --- |
| -56 | -14 | 7 | Left Superior Temporal/ Heschl's Gyrus | LSTG-TPJ | Auditory | 962 |
| 56 | -7 | 6 | Right Superior Temporal Gyrus | RSTG-TPJ | Auditory | 554 |
| 13 | -16 | -2 | Right Thalamus | RThal-Aud | Auditory | 26 |
| 14 | -2 | 8 | Right Thalamus. Caudate | RThal-Caud | Basal Ganglia | 669 |
| -14 | -3 | 7 | Left Thalamus. Caudate. | LThal-Caud | Basal Ganglia | 828 |
| -44 | 22 | 23 | Left Inferior Frontal Gyrus | LMFG | Basal Ganglia | 18 |
| 48 | 28 | 17 | Right Inferior Frontal Gyrus | RIFG-Gang | Basal Ganglia | 63 |
| -6 | -26 | -38 | Pons | PONS | Basal Ganglia | 32 |
| -30 | 23 | 49 | Left Middle Frontal eye Fields. | LMidFG-SupFG-FEF | LECN | 1501 |
| -40 | 48 | -1 | Left Inferior / Orbito Frontal Gyrus | lPFC-OrbFG | LECN | 437 |
| -42 | -63 | 46 | Left Superior / Inferior Parietal Sulcus. | LSPG-LOC-IPS | LECN | 2110 |
| -59 | -42 | -12 | Left Inferior / Middle Temporal Gyrus | LITG-MidTG-MT+ | LECN | 350 |
| 36 | -69 | -43 | Right Crus I | RCrus | LECN | 310 |
| -14 | -28 | 2 | Left Thalamus | LThal-Pariet | LECN | 8 |
| -49 | 25 | -4 | Inferior Frontal Gyrus | LIFG | Language | 652 |
| -52 | -1 | -22 | Left Middle Temporal Gyrus | LMTG | Language | 27 |
| -52 | -31 | -6 | Left Middle Temporal Gyrus. Angular Gyrus | LMTG-AngG | Language | 317 |
| -54 | -55 | 22 | Left Angular Gyrus. Inferior Parietal Lobule. | LAngG-IPL/TPJ | Language | 1420 |
| 49 | 28 | -10 | Right Inferior Frontal Gyrus | RIFG | Language | 58 |
| 55 | -43 | 10 | Right Angular Gyrus. Inferior Parietal Lobule. | RAngG-IPL | Language | 1106 |
| -22 | -80 | -35 | Left Crus I | LCrus | Language | 270 |
| 2 | -28 | 27 | Midcingulate / Posterior Cingulate Cortex | postCingC | Precuneus | 579 |
| 3 | -72 | 40 | Posterior Precuneus | posPCu | Precuneus | 1572 |
| -36 | -62 | 46 | Left Angular Gyrus | LAngG-PCu | Precuneus | 388 |
| 39 | -62 | 45 | Right Angular Gyrus | RAngG-PCu | Precuneus | 96 |
| 38 | 26 | 42 | Dorsomedial Prefrontal Cortex. | dmPFC-RSupFG-FEF | RECN | 2093 |
| 38 | 54 | 1 | Right Left Posterior Frontal Cortex | RlpFC | RECN | 356 |
| 48 | -54 | 47 | Right Inferior Parietal Gyrus. Intraparietal Sulcus | RIPG-LOC-IPS | RECN | 1873 |
| 5 | 37 | 46 | Right Superior Frontal Gyrus | RSFG-mPFC | RECN | 83 |
| -30 | -73 | -39 | Left Crus 1. Crus II. Lobule VI | Lcrus-LobVI | RECN | 2403 |
| 13 | 2 | 14 | Right Caudate | Rcau | RECN | 188 |
| -33 | -21 | 60 | Left Precentral / Postcentral Gyrus | LPreMotC-PreCenG | Sensorimotor | 1365 |
| 38 | -18 | 57 | Right Precentral / Postcentral Gyrus | RPreMotC-PreCenG | Sensorimotor | 1446 |
| 3 | -13 | 61 | Right Supplementary Motor Area | RSupplMotArea | Sensorimotor | 159 |
| -12 | -19 | -1 | Left Thalamus | LThal-Mot | Sensorimotor | 19 |
| -2 | -52 | -19 | Bilateral Lobule IV | Bil-Lob IV | Sensorimotor | 2015 |
| 12 | -20 | -4 | Right Thalamus | RThal-Mot | Sensorimotor | 20 |
| -27 | -1 | 54 | Left Middle Frontal Gyrus. Frontal Eye Fields | LMidFG-FEF | Visuospatial | 338 |
| -36 | -46 | 47 | Left Intraparietal Sulcus | LIPS | Visuospatial | 2020 |
| -47 | 13 | 27 | Left Frontal Operculum. Inferior Frontal Gyrus | LFOper | Visuospatial | 1105 |
| -49 | -65 | -6 | Left Middle Temporal Gyrus | LMTG-MT+ | Visuospatial | 93 |
| 28 | 2 | 54 | Right Middle Frontal Gyrus | RMidFG-FEF | Visuospatial | 97 |
| 37 | -47 | 48 | Right Inferior Parietal Lobule | RIPL | Visuospatial | 1193 |
| 49 | 12 | 28 | Right Frontal Operculum. | RFOper | Visuospatial | 326 |
| 50 | -59 | -11 | Right Middle Temporal Gyrus | RMTG-MT+ | Visuospatial | 76 |
| -27 | -72 | -52 | Left Lobule V111. | LLob V1-FEF | Visuospatial | 38 |
| 24 | -73 | -51 | Right Lobule VIII. | RLob VIII | Visuospatial | 131 |
| 34 | -70 | -26 | Right Lobule VI. Crus I | RLob VI-Crus | Visuospatial | 62 |
| -31 | 47 | 22 | Left Middle Frontal Gyrus | LMidFG-ACC | anterior Salience | 651 |
| -42 | 14 | -3 | Left Anterior Insula | LAntIns | anterior Salience | 305 |
| 0 | 17 | 47 | Anterior Cingulate Cortex. | ACingC | anterior Salience | 2887 |
| 28 | 46 | 26 | Right Middle Frontal Gyrus | RMidFG | anterior Salience | 470 |
| 43 | 15 | -1 | Right Anterior Insula | RAntIns | anterior Salience | 319 |
| -34 | -56 | -32 | Left Lobule VI. Crus I | LLob VI-Insula | anterior Salience | 95 |
| 36 | -58 | -32 | Right Lobule VI. Crus I | RLob VI | anterior Salience | 139 |
| -3 | 49 | 14 | Medial Prefrontal / Anterior Cingulate Cortex. | dMPFC-ACC | dorsal DMN | 5257 |
| -48 | -68 | 35 | Left Intraparietal Sulcus. Angular Gyrus | LIPS-AngG | dorsal DMN | 97 |
| 19 | 38 | 47 | Right Superior Frontal Gyrus | RSFG | dorsal DMN | 137 |
| 1 | -53 | 28 | Posterior Cingulate Cortex. Precuneus | PCC | dorsal DMN | 1555 |
| 2 | -15 | 36 | Midcingulate Cortex | MidCingC | dorsal DMN | 114 |
| 50 | -64 | 32 | Right Intraparietal Sulcus. Angular Gyrus | RIPS-AngG | dorsal DMN | 38 |
| -1 | -8 | 4 | Left and Right Thalamus | LRThal | dorsal DMN | 220 |
| -24 | -29 | -13 | Left Hippocampus | LHipp | dorsal DMN | 393 |
| 27 | -23 | -17 | Right Hippocampus | RHipp | dorsal DMN | 142 |
| -29 | -87 | -1 | Left Middle / Superior Occipital Gyrus | LMidOG-SupOG | high Visual | 868 |
| 32 | -85 | 0 | Right Middle /Superior Occipital Gyrus | RMidOG-SupOG | high Visual | 1679 |
| -39 | 35 | 30 | Left Middle Frontal Gyrus | LMidFG-INS | posterior Salience | 93 |
| -57 | -38 | 37 | Left Supramarginal/ Inferior Parietal Gyrus | LSupraMG-IPL | posterior Salience | 1205 |
| -8 | -52 | 61 | Left Precuneus | LPCu | posterior Salience | 98 |
| 12 | -28 | 45 | Right Midcingulate Cortex | RMidCinG | posterior Salience | 56 |
| 21 | -48 | 69 | Right Superior Parietal Gyrus. Precuneus | RSPG-PCu | posterior Salience | 133 |
| 59 | -32 | 36 | Right Supramarginal/ Inferior Parietal Gyrus | RSupramG-IPL | posterior Salience | 1002 |
| -12 | -21 | 5 | Left Thalamus | LThal-Insula | posterior Salience | 142 |
| -33 | -41 | -37 | Lobule VI I | Lob VI I | posterior Salience | 102 |
| -37 | -13 | -5 | Left Posterior Insula. Putamen | LPosIns-Put | posterior Salience | 114 |
| 13 | -14 | 9 | Right Thalamus | RThal-Insula | posterior Salience | 63 |
| 36 | -43 | -40 | Lobule VI II | Lob VI II | posterior Salience | 13 |
| 40 | -6 | -9 | Right Posterior Insula | RPosIns | posterior Salience | 134 |
| 0 | -74 | 11 | Calcarine Sulcus | CalcS | primary Visual | 1116 |
| -18 | -24 | -4 | Left Thalamus | LThal-V1 | primary Visual | 4 |
| -12 | -58 | 15 | Left Retrosplenial and Posterior Cingulate Cortex | LRetC-PCC | ventral DMN | 462 |
| -24 | 12 | 55 | Left Middle Frontal Gyrus | LMidFG | ventral DMN | 405 |
| -28 | -37 | -15 | Left Parahippocampal Gyrus | LParahG | ventral DMN | 134 |
| -36 | -81 | 32 | Left Middle Occipital Gyrus | LMOG | ventral DMN | 491 |
| 13 | -53 | 14 | Right Retrosplenial and Posterior Cingulate Cortex | RRetC-PCC | ventral DMN | 590 |
| 1 | -57 | 54 | Anterior Precuneus | antPCu | ventral DMN | 1921 |
| 26 | 26 | 45 | Right Superior / Middle Frontal Gyrus | RSFG-MidFG | ventral DMN | 399 |
| 28 | -33 | -19 | Right Parahippocampal Gyrus | RParahG | ventral DMN | 90 |
| 43 | -74 | 32 | Right Angular /Middle Occipital Gyrus | RAngG-MidOG | ventral DMN | 752 |
| 15 | -46 | -53 | Right Lobule IX | Rlob IX | ventral DMN | 81 |

Table S1: List of selected ROIs. Coordinates (x,y,z) of ROIs used for functional connectivity analysis, according to MNI atlas.
